# Flat-Lens Focusing of Electron Beams in Graphene

**DOI:** 10.1038/srep33522

**Published:** 2016-09-15

**Authors:** Yang Tang, Xiyuan Cao, Ran Guo, Yanyan Zhang, Zhiyuan Che, Fouodji T. Yannick, Weiping Zhang, Junjie Du

**Affiliations:** 1 Quantum Institute for Light and Atoms, Department of Physics, East China Normal University, Shanghai 200062, China

## Abstract

Coupling electron beams carrying information into electronic units is fundamental in microelectronics. This requires precision manipulation of electron beams through a coupler with a good focusing ability. In graphene, the focusing of wide electron beams has been successfully demonstrated by a circular p-n junction. However, it is not favorable for information coupling since the focal length is so small that the focal spot locates inside the circular gated region, rather than in the background region. Here, we demonstrate that an array of gate-defined quantum dots, which has gradually changing lattice spacing in the direction transverse to propagation, can focus electrons outside itself, providing a possibility to make a coupler in graphene. The focusing effect can be understood as due to the gradient change of effective refractive indices, which are defined by the local energy band in a periodic potential. The strong focusing can be achieved by suitably choosing the lattice gradient and the layer number in the incident direction, offering an effective solution to precision manipulation of electron beams with wide electron energy range and high angular tolerance.

Graphene has exceptional electrical properties, including high electrical conductivity, high carrier mobility, ballistic transport, etc.^1^, which provide a basis for its potential applications in electronics. Transistors^2^,^3^,^4^,^5^,^6^, transparent conductive coatings^7^,^8^, interconnects and so on, stand a chance of benefiting from the development of graphene. The exceptional mechanical^9^,^10^, thermal^11^, optical^12^ and chemical^13^ properties of graphene endow these electronic elements unique advantages. For instance, graphene-based transparent conductive coatings can be used to design rollable electronic paper, foldable organic light-emitting diodes and touch screen displays. However, before the superior properties of graphene can be translated into applications in electronics, an important issue to be resolved is how to couple electron beams, information carrier, into electronic units. This could be implemented by an interface device which functions as a coupler. In the process of coupling, precision manipulation of electron beams requires couplers to focus wide electron beams into small spots. Though a focusing effect has been demonstrated by using a circular p-n junction in monolayer graphene^14^,^15^,^16^,^17^, the structure is not suitable to act as a coupler since its focal spot locates inside the circular gated region due to the small focal length. In addition, the focal imaging was realized by a planar p-n junction^18^ which, however, fails to focus a parallel electron beam. Therefore, it is extremely appealing to develop a focusing device suitable to couple electron beams into electronic units.

Both the focusing and the focal imaging mentioned above are achieved based on parallels between optics and electronics in monolayer graphene. Quantum Fabry-Pérot interferences for electrons also has been realized in graphene^19^. The analogies are due to a linear energy dispersion near the Dirac point in monolayer graphene, in which low-energy quasiparticles behave like massless relativistic Dirac fermions and is subjected to Dirac equation^1^. The circular p-n junction functions as a curved lens and the focusing effect can be understood by a semiclassical picture from catastrophe optics. While the planar p-n junction possesses the properties of optical metamaterials^20^,^21^ and electron waves arriving it will exhibit negative refraction behavior^18^. Namely, the direction of the group velocity is opposite to that of wave vector in the valence band. When a point-like source is placed in front of the p-n junction, there will be a focal image inside it or on the transmission side and this structure is called a Veselago electronic Lens. Quantum electron optics based on the analogies between optics and electronics offers almost unlimited possibilities for the development of graphene electronics^15^,^22^,^23^,^24^,^25^.

In the past decades, the exploitation for artificial optical structures has largely stimulated the development of photonics and optics. Photonic crystals, as a typical example, have shown a powerful ability in manipulating the flow of light for their abundant frequency-wavevector dispersion relation^26^. Electrons in a periodic potential, which is realized by periodically arrayed gate-defined quantum dots, might be very like light in periodic dielectric in photonic crystals. So it is important and meaningful to apply some conceptions in photonic crystals to graphene for manipulation of propagation of electron waves. Some effects such as quasibound states, the resonant transport and the defect states, similar to those in photonic crystals, have been studied in graphene quantum dot superlattices^27^,^28^,^29^,^30^,^31^. Here, we calculate the energy-momentum dispersion relation of electrons in a periodic potential in graphene, and on this basis, design a graded array acting as a flat lens to focus a parallel electron beam. Similar to photonic crystals^32^,^33^, we can also define an effective refractive index (ERI) in periodic potentials for electron waves by its energy-momentum dispersion relation. The gradually changing ERI, which is realized by the gradually changing lattice spacing, will result in the focusing of a parallel electron beam. We will see that the focal spot locates outside the array and a coupler could be designed based on the structure.

## Results

**Figure 1:**
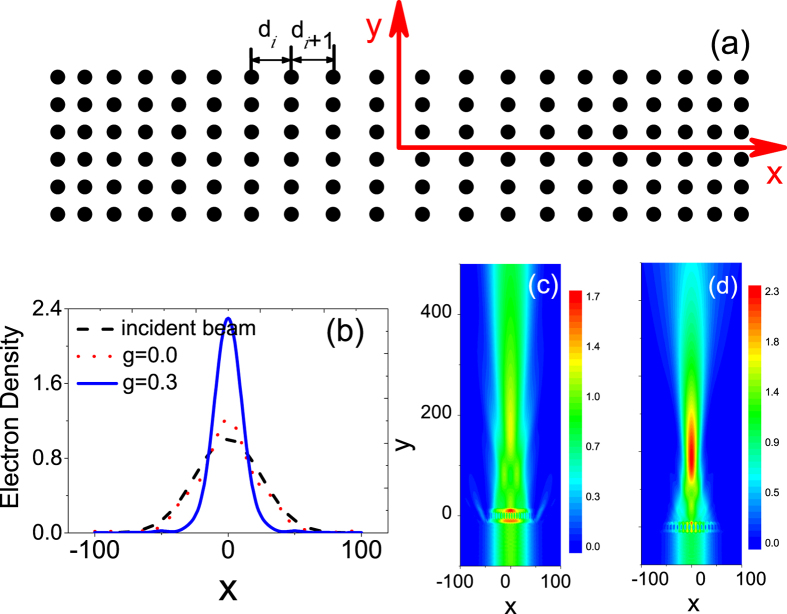
(**a**) The schematic representation of the array with the gradually changing lattice constant. (**b**) Electron density along the x-direction line passing through the focal spot in three cases. Electron density distribution for the array without the lattice gradient (**c**) and with the gradient increment g = 0.3 (**d**). The energy of the incident electron is *E* = 0.5 and the incident electron beam has unit electron density at the beam center. The obvious focusing effect can be observed in Fig. 1(d) when the array has the lattice gradient.

In the scattering problem of electron waves with energies *E* close to the Dirac point, the Hamiltonian can be written as


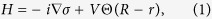


where Θ(*R* − *r*) is the Heaviside function with *R* the radius of the circular potential, *V* is the applied constant bias of the gated region and *σ* = (*σ*_*x*_, *σ*_*y*_) are Pauli matrices. We employ reduced units, i.e., *ħ* = 1 and the Fermi velocity *v*_*F*_ = 1 throughout. The focusing is realized with an array of gate-defined quantum dots which share an identical radius *R* = 1 in graphene and are applied a constant bias *V* = 5. Here all quantities with displacement dimension are in unit of the radius *R* since it is set to be 1. At the same time, the incident electron beam has unit electron density at the beam center for the convenience of comparison when the electron density is plotted in all figures. The unmodified structure is a square-lattice crystal with lattice constant *d* = 5. In order to achieve the focusing effect, a lattice gradient in the transverse direction (perpendicular to the incident wave vector) is added to the original. We study an *n*_*t*_ × *n*_*l*_ array which is illuminated by a Gaussian beam along the +*y* direction, where *n*_*t*_ and *n*_*l*_ denote the layer number of quantum dots in the transverse (*x*) and longitudinal (*y*) direction, respectively. In the two edges of the array, there is the equal lattice spacing in the *x* and *y* direction and then the lattice spacing in the *x* direction will linearly increase from the two edges to the center with the increment *g* = *d*_*i*+1_ − *d*_*i*_, as is schematically shown in Fig. 1(a).

The focusing effect is simulated in an array with *n*_*t*_ = 20 and *n*_*l*_ = 6 and the energy of the incident electron *E* = *k* = 0.5 in Fig. 1. The spatial distribution of electron density is plotted in Fig. 1(c,d) for the array without (with) the lattice gradient. The obvious focusing effect can be seen in Fig. 1(d) when the lattice gradient exists. While the propagation of electron waves is hardly affected by the unmodified square-lattice array, as shown in Fig. 1(c). In Fig. 1(b), we plot the electron density along the x-directional line across the focal spot (in Fig. 1(d)) for the cases in Fig. 1(c,d) and for the initial incident wave, respectively. One can see that for the non-gradient case, both the intensity and the distribution of the electron density only have a negligibly change, compared to the incident wave. When the array has gradient, the electron density at the focal spot increases by 2.29 times and correspondingly the waist radius obviously decreases. The comparison indicates that the focusing effect is realized due to the existence of the lattice gradient. In Fig. 1(d), the focal spot lies outside the array, showing that the structure can be used as a coupler. When considering the practical realization, one can choose a typical radius *R* = 5 nm, and thus the longitudinal length of the array in Fig. 1(d) is 125 nm with the energy *E* = 65.6 meV and the bias *V* = 656 meV.

## Discussion

**Figure 2:**
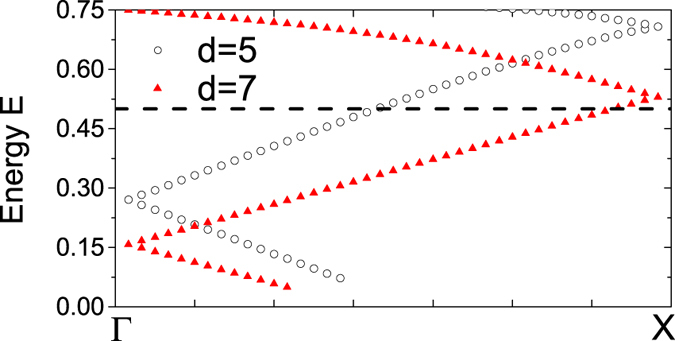
Energy band structure of electrons for two different square-lattice arrays of quantum dots in graphene with lattice constant *d* = 5 (**a**) and *d* = 7 (**b**). The black dashed line indicates the energy of the incident electrons *E* = 0.5. Both dispersion bands are approximately the straight line for the energy *E* ranging from 0.3 to 0.5, and their slopes respectively define an effective refractive index *n*_*eff*_.

The focusing of electron waves can be similarly understood as that of light through optical lenses. In optics, an optical system for focusing satisfies the principle of equal optical path according to Fermat’s principle^34^. Since optical path is determined by the spatial distance light travels and the refractive index, the principle of equal optical path requires that one of both must vary when light is focused by a converging lens. For conventional convex lenses, the propagation distance will continuously change due to its curved surface. But for a flat lens, the principle of equal optical path has to depend on the change of refractive index. Similarly, we also need to define an ERI for electron waves in quantum-dot arrays in graphene by using the concept of the local energy band. To this end, we respectively calculate the energy band structure of electrons for two periodic structures of quantum dots with square lattice in graphene in Fig. 2. The black (circle) line corresponds to the array with lattice constant *d* = 5 while the red (triangle) line *d* = 7. One can see that the energy band exhibits a noticeable variation when the lattice constant is changed. For the dispersion relation of light in photonic crystals, we have *dω*/*dk* ∝ 1/*n* where *n* is the ERI and thus the slope of the optical band is related with the ERI^35^,^36^. Correspondingly, we define an ERI *n*_*eff*_ for electron waves in graphene with *dE*/*dk* ∝ 1/*n*_*eff*_ based on the same principle. In Fig. 2 each dispersion band is approximately a straight line for the energy *E* ranging from 0.3 to 0.5, so their different slopes illustrate that the larger lattice constant produces the larger ERI. This means that the gradient of lattice constant will lead to the gradient of the ERI *n*_*eff*_. Thus the array probably satisfies the requirement of the principle of equal optical path due to the ERI gradient and has ability to focus electron waves.

**Figure 3:**
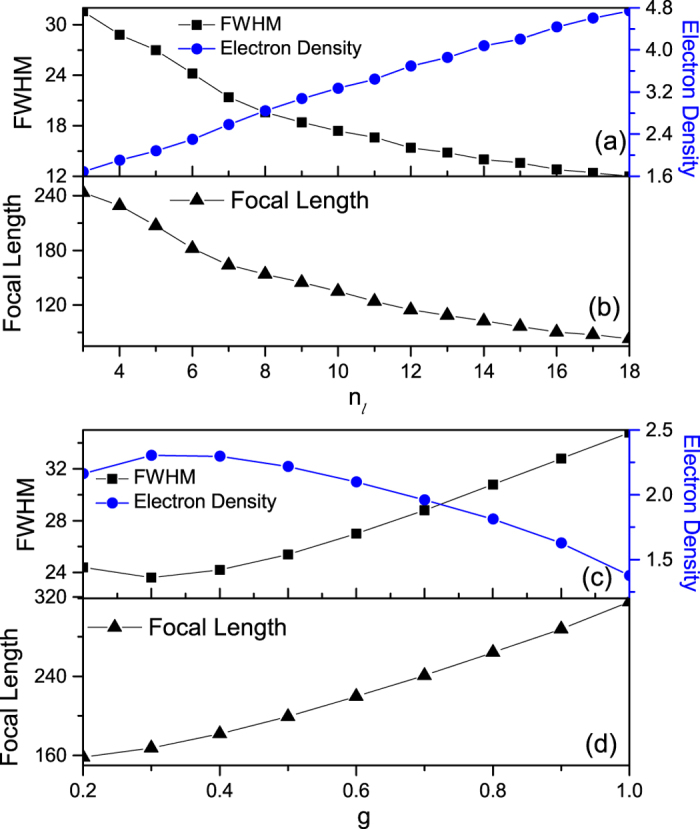
Electron density at focus and FWHM (**a**) and focal length (**b**) versus the layer number in the longitudinal direction *n*_*l*_ with *g* = 0.4 fixed. Electron density at focus and FWHM (**c**) and focal length (**d**) versus the lattice increment *g* with *n*_*l*_ = 6 fixed. The energy of the incident electron is *E* = 0.5 and *n*_*t*_ = 20.

**Figure 4:**
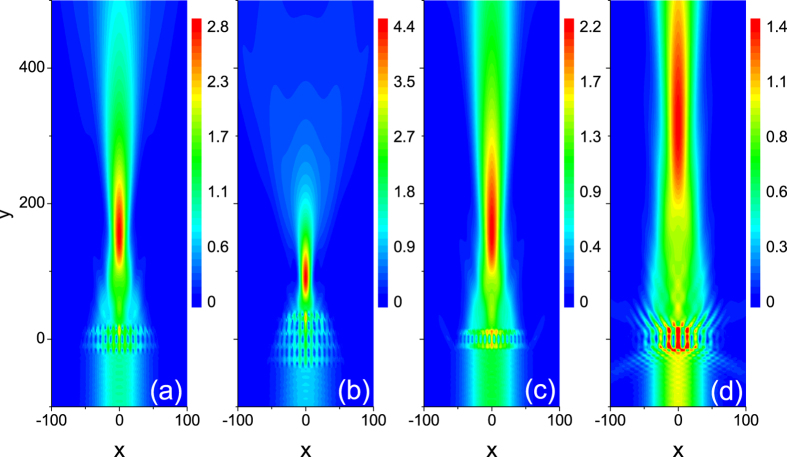
Electron density distribution for the array with *n*_*l*_ = 8 (**a**) and *n*_*l*_ = 16 (**b**) when the lattice increment *g* = 0.4 remains unchanged. Electron density distribution for the array with the lattice increment *g* = 0.2 (**c**) and *g* = 1.0 (**d**) when *n*_*l*_ = 6 remains unchanged. The energy of the incident electron is *E* = 0.5 and *n*_*t*_ = 20.

Just like an optical lens, the focussing property of electron waves is also related with the geometrical property of the array, such as its thickness and the gradient. We first keep the increment *g* = 0.4 and *n*_*t*_ = 20 unchanged and see the effect of the thickness, i.e., the layer number in the longitudinal direction *n*_*l*_, on focal length and the electron density at focal spot. Figure 3(a) displays the electron density at focal plane which increases with *n*_*l*_ enhancing from 3 to 18. It seems that the more the layer number the electron wave travels is, the more strongly it is focused. In order to verify the assumption, we plot the full width at half maximum (FWHM) along the x-directional line at focal plane to characterize the size of focal spots in Fig. 3(a). We see that the FWHM decreases with *n*_*l*_ increasing, showing that the electron wave is better focused with the *n*_*l*_ increasing. Figure 3(b) shows that the focal length becomes small as *n*_*l*_ increases. Namely, the focus spot is more and more close to the array with the layer number increasing. This means that the array with larger thickness is more like an optical converging lens with smaller radius of curvature. One can also note that the focal spot always locates outside the array for arbitrary *n*_*l*_, meeting the requirement of a coupler. To illustrate these features of the flat lens, we plot the electron density distribution in a large spatial region in Fig. 4(a,b) for *n*_*l*_ = 8 and *n*_*l*_ = 16, respectively. We can find more details about the focusing from them. First, the electron wave is hardly reflected by the arrays. The fact that all of waves can arrive at the transmission side ensures that our design has a high focusing efficiency. Second, a strong focusing shown in Fig. 4(b) can be realized with the layer number *n*_*l*_ larger than 16.

The gradient is another factor that makes an effect on the focusing property. In Fig. 3(c,d), the lattice increment *g* is changed from 0.2 to 1.0 and the layer number in the longitudinal direction *n*_*l*_ = 6 and in the transverse direction *n*_*t*_ = 20 remains unchanged. One can see that the electron density at focal spot tends to achieve the maximum value when the increment *g* is in the vicinity of 0.3 and the FWHM has the minimum value at the same time, implying that a good focusing occurs. This demonstrates that the increment *g* = 0.3 is an optimal value of the gradient for the array to product the focusing behavior. To understand the existence of the optimal value, we plot the electron density distribution in a spatial region for the arrays with the increment *g* = 0.2 and *g* = 1.0 in Fig. 4(c,d), respectively. The obvious electron scattering in other directions can be seen in Fig. 4(d), preventing a part of electron waves being transmitted and focused. Since the change of the increment will result into the varying ERI according to the energy band plot in Fig. 2, we can owe the degradation of the focusing to the inappropriate ERI when *g* is far away from 0.3. In the language of light, too small change of ERI is not enough to deflect the wave, while too large change will lead to the scattering of waves in other directions (as the case in Fig. 4(d)). In other words, the principle of equal optical path will be difficult to be satisfied due to the inappropriate ERI. We also note that although the FWHM has an optimal value, the focal length in Fig. 3(d) always becomes larger when the gradient enhances. Therefore the array with a small gradient is like an optical lens with a small radius of curvature. Again, the array has the enough large focal length for arbitrary *g* to make sure that the focal spot locates outside itself.

Based on the above analysis for the focusing properties, we can realize the strong focusing by designing an array with *n*_*l*_ larger than 16 and *g* in the vicinity of 0.3, as shown in Fig. 4(b). The electron density at focal spot is 4.4 times as large as that of the incident Gaussian wave, and the FWHM is 21% smaller than that of the incident wave. In addition, we can also achieve the desirable focal length by suitably choosing the longitudinal layer number *n*_*l*_ and the gradient.

**Figure 5:**
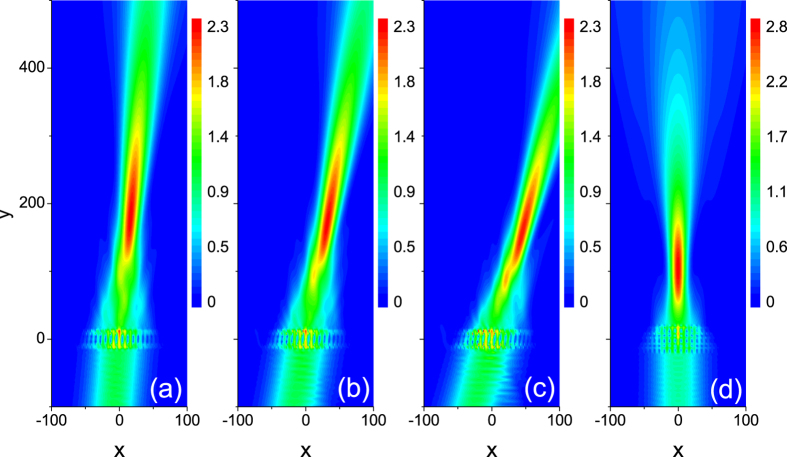
Electron density distributions for the incident angle *θ*_*i*_ = 5° (**a**), *θ*_*i*_ = 10° (**b**) and *θ*_*i*_ = 15° (**c**) with *g* = 0.4 and *n*_*l*_ = 6. The energy of the incident electron is *E* = 0.5. (**d**) Electron density distribution for the same array as in Fig. 4(a) when the electron energy is changed from *E* = 0.5 to 0.4.

Until now, we only study the focusing of electron waves for the normal incidence case. When considering practical applications as a coupler in graphene-based microelectronics, we expect that the focusing is also robust for non-normal incident angles. In Fig. 5(a–c), we display the focusing effect by an array with *n*_*l*_ = 6 and *g* = 0.4 when the angle of incidence is *θ*_*i*_ = 5°, 10°, and 15°, respectively. One can see that electron density at focal spot and the FWHM do not obviously change with the angle of incidence enhancing. It shows that the focusing effect has a high angle tolerance.

Once a quantum-dot array with the definite layer number and gradient is patterned in graphene, it is not practical to change its geometric structure. Therefore, it is expected that electron waves with different energy can be focused by it. For the same array as in Fig. 4(a), the electron density distribution displayed in Fig. 5(d) shows that the wave with electron energy *E* = 0.4 still can be well focused. The further calculation demonstrates that such an array can focus waves with electron energy *E* ranging from 0.32 to 0.5. The energy band plot in Fig. 2 clearly shows that the ERI has only a slight variation in a wide energy range with nearly the same slope. Therefore, the focusing by the graded array can occur in this wide range.

In summary, electron waves in graphene can be focused using a quantum dot array with gradually changing lattice constant in the transverse direction. With the total transmission of electron waves after passing through the array, the strong focusing can be even achieved by choosing the appropriate gradient and the layer number in the longitudinal direction. The large focal length ensures that focal spot lies outside the array and the structure can be used as a coupler. The design paves a new way for precision manipulation of electron beams in the graphene electronics with the high angular tolerance and the wide energy range.

## Methods

The simulation is done by first solving the scattering problem of a single quantum dot based on Mie scattering method in graphene^14^,^16^,^37^. The incident and scattered waves are expanded as follows:


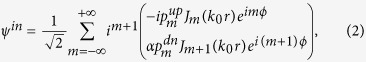



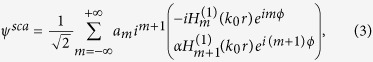


where 
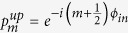
, 
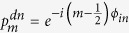
, *J*_*m*_ and 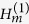 are, respectively, the *m*th order Bessel function and Hankel function of the first kind, *ϕ*_*in*_ is the angle of incidence. Similarly, the inner field inside the rod can be written as


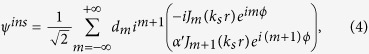


where *k*_0_ and *k*_*s*_ is respectively the wave vector in the background region and the gated region and *k*_*s*_ = *nk*_0_ with the refractive index *n* = |*V* − *E*|/*E*. After imposing the boundary condition at the surface of the circular potential with its radius R, the scattering coefficient *a*_*m*_ is given


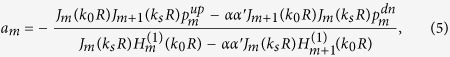



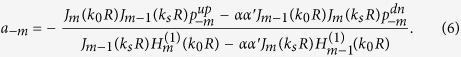


We can also express the relation between the scattering coefficients *a* and the incident coefficients 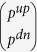 in Eqs (5) and (6) by a scattering matrix S.

In a system of multiple gated potentials, the incident wave that strikes the surface of potential j consists of two parts: (1) the initial incident wave and (2) the scattered waves of all other potentials according to multiple scattering theory^38^ (also known in solid-state physics as the K. K. R. method^39^,^40^). Thus it can be written as 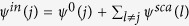. By the translational addition theorem, the scattered wave from any other potential *l* (with *l* ≠ *j*) can be expanded as follows,


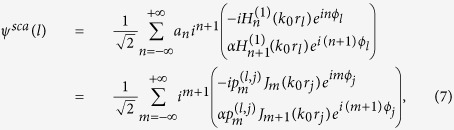


where


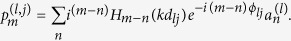


Substituting (7) into *a*^(*j*)^ = *Sp*^(*j*)^, one will yield a set of linear equations that contains the iterative scattering coefficients. With the equations, we can compute the scattering field at any position and the energy band structure.

In practical numerical calculation, the series expansion of Eqs (2, 3, 4) and (7) has to be truncated at some *n* = *n*_*c*_. The resultant error incurred due to truncation is assumed to be insignificant. In our problem, the minimum *n*_*c*_ should be set to be 5 in order to guarantee convergence. The calculation for any system of multiple gated potentials in this paper usually can be completed within several minutes in a personal computer system.

## Additional Information

**How to cite this article**: Tang, Y. *et al*. Flat-Lens Focusing of Electron Beams in Graphene. *Sci. Rep.* **6**, 33522; doi: 10.1038/srep33522 (2016).
